# *Cereus jamacaru* D.C. Hydroalcoholic Extract Promotes Anti-Cytotoxic and Antitumor Activity

**DOI:** 10.3390/ph11040130

**Published:** 2018-11-23

**Authors:** Jean Carlos Vencioneck Dutra, Jean Moisés Ferreira, Paula Roberta Costalonga Pereira, Judá Ben-Hur de Oliveira, Suiany Vitorino Gervásio, Mirieli Bernardes Xavier, Mainã Mantovanelli da Mota, Anny Carolyne da Luz, Irany Rodrigues Pretti, Hildegardo Seibert França, Claudia Masrouah Jamal, Maria do Carmo Pimentel Batitucci

**Affiliations:** 1 Laboratório de Genética Vegetal e Toxicológica, Departamento de Ciências Biológicas, Universidade Federal do Espírito Santo, Vitória 29075-910, Brazil; paula_costalonga@hotmail.com (P.R.C.P.); judex_21@hotmail.com (J.B.-H.d.O.); suianygervasio@gmail.com (S.V.G.); mirieli.bernardes@gmail.com (M.B.X.); maina.mantovanelli@gmail.com (M.M.d.M.); annydaluz@gmail.com (A.C.d.L.); iranyrpretti@gmail.com (I.R.P.); docarmo_batitucci@yahoo.com.br (M.d.C.P.B.); 2 Laboratório de Biologia Molecular e Expressão Gênica, Departamento de Ciências Biológicas, Universidade Federal de Alagoas, Arapiraca 57309-005, Brazil; jean.moises@hotmail.com; 3 Instituto Federal do Espírito Santo, Vila Velha 29106-010, Brazil; hildegardo.franca@ifes.edu.br; 4 Laboratório de Química de Produtos Naturais, Departamento de Ciências Farmacêuticas—Universidade Federal do Espírito Santo, Vitória 29040-090, Brazil; cmjamal@gmail.com

**Keywords:** mandacaru, phytochemistry, antioxidant activity, MTT assay, sarcoma 180 antitumor

## Abstract

*Cereus jamacaru* D.C. (mandacaru) is a cactus used as food and in the traditional medicine. In the present study, hydroalcoholic extract of *C. jamacaru* was evaluated for its chemical composition, antioxidant activity, cytotoxic and anti-cytotoxic effects in human lymphocytes and sarcoma 180 cells *in vitro* by MTT assay and antitumoral, mutagenic and cytotoxic effects on mice sarcoma-induced *in vivo*. Phytochemical characterization showed positive reactions for coumarin, flavanol and tyramine and total flavonoid content of 0.51 µg/mL. *C. jamacaru* showed antioxidant activity following DPPH (EC_50_ = 427.74 µg/mL), ABTS (EC_50_ = 270.57 µg/mL) and Fe^2+^ chelating ions assays (EC_50_ = 41.18 µg/mL). *C. jamacaru* induced significant decrease of sarcoma 180 viability at 24 h and 48 h of treatment, did not induce cytotoxicity in human lymphocytes and inhibits the cytotoxicity of cisplatin *in vitro*. Following *in vivo* assays, *C. jamacaru* promoted tumor reduction (86.07% of tumor inhibition), without inducing mutagenic or cytotoxic damage on mice blood cells. We propose that phenolic and alkaloid compounds in the extract are related to antioxidant activity, increasing its ability in metal chelating activity and promoting anti-cytotoxic activity against cisplatin, as well as these compounds may act on the cell cycle of the tumor cells *in vitro* and *in vivo*, leading to anticancer effects and tumor reduction.

## 1. Introduction

Brazil presents the major flora diversity of the world, exclusives biomes and a great number of vegetal species adapted to arid regions [1], such as cacti. *Cereus jamacaru* D.C., commonly known as “mandacaru,” is a native Brazilian cactaceae that occur naturally in Caatinga biome and is described as a source of food and medicinal products used in the treatment of urinary infection, kidney inflammation and rheumatism [2].

Natural products may contain non-fully identified compounds that present biological activities, inducing minimal side effects in biological systems and purchased at a relatively low cost [3]. Additionally, medicinal plants used as food, such as cacti, may have compounds such as flavonoids and alkaloids and exhibit strong antioxidant and anticancer effects [4,5,6]. In that way, cactus plants used for humans may exhibit wide variety of phytocompounds, such as phenolic and nitrogen compounds, which have been related to their biological activities [7,8,9].

*In vitro* and *in vivo* assays using human and rodent cells have been used to access the cytotoxicity and mutagenicity of many substances, such as natural or semi-synthetic products, as well as their effects on DNA [10,11,12]. *In vitro* techniques provide multiple and complementary measures of cytotoxicity [13] and can be used to evaluate the effect of natural products and chemotherapeutic compounds on direct contact with cells [14,15]. In addition, *in vivo* studies, such as those using rodents, have been used to complement *in vitro* studies and may help to understand the effect of metabolization and the toxicological effects of various compounds [11], as well as their potential use in the treatment of cancers.

To our knowledge, the effects of *C. jamacaru* consumption and its possible antioxidant, anti-cytotoxic and anticancer activities are unclear and not well documented in the literature. Thus, the aim of this study was to evaluate the *C. jamacaru* extract chemical composition, antioxidant activity, cytotoxic and anti-cytotoxic effects in human lymphocytes and sarcoma 180 cells *in vitro* and antitumoral, mutagenic and cytotoxic effects *in vivo* mice sarcoma-induced.

## 2. Material and Methods

### 2.1. Chemicals

Ficoll^®^ Paque Plus (Sigma–Aldrich, Missouri, United States); RPMI 1640 culture medium (Cultilab, Campinas, Brazil); fetal calf serum (Gibco, Miami, United States); Cisplatin (Fauldcispla^®^, Libbs, São Paulo, Brazil); 2,2-diphenyl-1-picrylhydrazyl (Sigma–Aldrich, Missouri, United States); 2,2′-azino-bis-(-3-ethylbenzothiazoline-6-sulfonic acid) (Sigma–Aldrich, Missouri, United States); 3-(4,5-dimethyl2-thiazolyl)-2,5-diphenyl-2H-tetrazolium bromide (Sigma–Aldrich, Missouri, United States).

### 2.2. Plant Material

*C. jamacaru* cladodes were collected at Alagoas state, Northeast of Brazil (9°40′44.7′′ S, 36°41′21.9′′ W), in September 2016. A voucher specimen was deposited in the Herbarium of Universidade Federal do Espírito Santo—VIES. The thorns of plant material were removed with a blade, the cladodes were sliced and 485 g of the fragmented material was oven dried at 50 °C for 24 h.

### 2.3. Hydroalcoholic Extract

Dried plant material was powdered, macerated in EtOH/H_2_O (70:30 *v*/*v*) solution (dry plant: EtOH/H_2_O—1:5 *w*/*v*) at room temperature (25–30 °C), protected from the light, for five days, filtered and evaporated under reduced pressure at 60 °C to obtain the crude extract of *C. jamacaru*. The extract was stored at 6–10 °C and protected from the light until use. The yield of the extract was calculated by the formula:

Total extract yield (%) = (FM/IM) × 100

where “FM” = final mass of dry extract (g); “IM” = initial mass of dry plant (g).

### 2.4. Phytochemistry Analysis

#### 2.4.1. Preliminary Phytochemistry

In order to identify secondary metabolites, preliminary phytochemical prospection was performed using *C. jamacaru* crude extract. Tests for coumarins, flavonoids, alkaloids, naphthoquinones, saponin, steroids, tannins and triterpenoids were conducted according to the procedures described in the literature [16].

#### 2.4.2. Thin Layer Chromatography

Identification of tyramine in *C. jamacaru* extract was performed by thin layer chromatography (TLC), as described by Davet et al. [7].

#### 2.4.3. Flavonoid Content

Total flavonoid content of *C. jamacaru* extract was measured by the colorimetric method described for Zhishen et al. [17], with minor modifications. The absorbance at 430 nm was detected by ELISA reader and the experiment was performed in triplicate. Methanolic dilutions series of rutin were prepared and assayed. The amount of flavonoid in extract was expressed in milligram of flavonoid equivalent to rutin per gram of dry matter of extract.

### 2.5. Antioxidant Activity

#### 2.5.1. DPPH

Antioxidant activity of *C. jamacaru* extract was evaluated by the radical reduction method, DPPH• (2,2-diphenyl-1-picrylhydrazyl), which fixing an H• leads to a decrease in absorbance [18]. The absorbance was taken by ELISA reader at 517 nm and the test was performed in triplicate. The reduction percentage of the DPPH radical was calculated by the following formula:

% inhibition = [(AbsControl − AbsSample)/AbsControl] × 100

where “% inhibition” is the percentage of inhibition capacity of DPPH•; “AbsControl” is the absorbance of DPPH• reaction control; and “AbsSample” is the absorbance of the sample.

#### 2.5.2. ABTS

Total antioxidant activity of *C. jamacaru* extract was measured by capturing method 2,2′- azino-bis-(-3-ethylbenzothiazoline-6-sulfonic acid) (ABTS), ABTS•^+^ radical [19]. The absorbance was taken by ELISA reader at 734 nm and experiment was performed in triplicate. The percentage of scavenging inhibition capacity of ABTS•^+^ of *C. jamacaru* extract was calculated by the following equation:

% inhibition = [(AbsControl − AbsSample)/AbsControl] × 100

where “% inhibition” is the percentage of scavenging inhibition capacity of ABTS•^+^; “AbsControl” is the absorbance of ABTS•^+^ reaction control; and “AbsSample” is the absorbance of the sample.

#### 2.5.3. Fe^2+^ Chelation Ions

Ferrous ions (Fe^2+^) chelating activity was measured by the inhibition of ferrous–ferrozine complex formation after treatment with *C. jamacaru* extract [20]. The absorbance was measured by the ELISA reader at 562 nm. The percentage of ferrous ion chelating effect was calculated using the following equation:

% chelating effect = [(AbsControl/AbsSample)/AbsControl] × 100

where “% chelating effect” is the percentage of ferrous ions (Fe^2+^) chelating effect; “AbsControl” is the absorbance of Fe^2+^ reaction control; and “AbsSample” is the absorbance of the sample.

### 2.6. In Vitro Cell Assays

#### 2.6.1. Human Lymphocytes

Human lymphocytes were obtained from the peripheral blood sample of a healthy non-smoking volunteer with informed consent, aged between 20 and 30 years, with no history of recent disease, exposure to radiation or drug use and no alcohol ingestion thirty days before blood donating. The lymphocytes were isolated by the traditional method on Ficoll^®^ Paque Plus gradient, as recommended by manufacturer, with minimal modifications. All protocols were approved by the Research Ethical Committee of Universidade Federal do Espírito Santo (certificate 2.333.879). Human lymphocytes were plated in 96-well plates with 2.10^5^ cells in each well. Cells from the control group were not treated and cisplatin group cells received cisplatin at 50.0 μg/mL. To evaluate cytotoxicity, human lymphocytes cells received *C. jamacaru* extract diluted with water at 10.0, 50.0 or 100.0 μg/mL. The cells were cultured with *C. jamacaru* extract for 24 h or 48 h. In order to allow the evaluation of the anti-cytotoxicity, human lymphocytes were treated with *C. jamacaru* extract more cisplatin following the protocols of pre-treatment, simultaneous treatment and post-treatment. In the pre-treatment protocol, the cells were previously treated with *C. jamacaru* extract diluted with water at 10.0, 50.0 or 100 μg/mL and 24 h after was added cisplatin at 50.0 μg/mL. Cells in the simultaneous protocol received *C. jamacaru* extract at 10.0, 50.0 or 100.0 μg/mL and cisplatin at 50.0 μg/mL simultaneously. In the post-treatment protocol the cells previously received cisplatin at 50.0 μg/mL and 24 h after the cell were treated with *C. jamacaru* extract at 10.0, 50.0 or 100.0 μg/mL.

The percentage of cytotoxic damage reduction was calculated using the adapted formula [21]:

$$\%\;\mathrm{Reduction}=\frac{\left(\%\;\mathrm{cell}\;\mathrm{viability}\;\mathrm{in}\;A-\%\;\mathrm{cell}\;\mathrm{viability}\;\mathrm{in}\;B\right)}{\left(\%\;\mathrm{cell}\;\mathrm{viability}\;\mathrm{in}\;A-\%\;\mathrm{cell}\;\mathrm{viability}\;\mathrm{in}\;C\right)}\times 100$$

where “A” is the cell group treated with cisplatin; “B” is the cell group treated with *C. jamacaru* extract more cisplatin; and “C” is the control group of cells.

#### 2.6.2. Sarcoma 180

Sarcoma 180 cells (murine sarcoma) were acquired from Banco de Células do Rio de Janeiro and all protocols were approved by the Research Ethical Committee of Universidade Federal do Espírito Santo (certificate 89/2015). Sarcoma 180 cells were plated in 96-well plates with 2.10^5^ cells in each well. Control group cells were untreated and cisplatin group cells were treated with cisplatin at 50.0 μg/mL. Treated cells received *C. jamacaru* extract diluted with water at 10.0, 50.0 or 100.0 μg/mL. Cells were cultured with *C. jamacaru* extract for 24 h or 48 h to evaluate its antiproliferative effect.

#### 2.6.3. Cell Culturing Methods

Cells were cultured with RPMI 1640 culture medium, supplemented with antibiotic gentamicin (50.0 mg/L) and antimycotic amphotericin B (2.0 mg/L) and 10% of fetal calf serum at 37 °C, 5% of CO_2_ saturation and humid atmosphere. Cells were cultured under these conditions 24 h before starting the treatments. 24 h after the last treatment, MTT assay was used to determine cell viability.

#### 2.6.4. MTT Assay

The 3-(4,5-dimethyl-2-thiazolyl)-2,5-diphenyl-2H-tetrazolium bromide (MTT) assay was performed to evaluate cell viability. After treatment, the plates were centrifuged at 860 rcf for 10 min, the supernatant discarded and 20 μL of MTT at 5 mg/mL were added to each well. 3 h later, the plates were centrifuged at 860 rcf for 5 min, the supernatant was discarded, 100 μL of DMSO was added and absorbance was detected in ELISA reader at 590 nm. The experiment was performed in triplicate and the results were expressed as relative percentage of cell viability in comparison to control.

### 2.7. In Vivo Mice Antitumor

#### 2.7.1. Animals and Sarcoma Induction

Twenty albino male mice *Swiss* strain (*Mus musculus*) (*n* = 20), aged 6 to 8 weeks and approximately 40 g of body weight (b.w.) were supplied by the biotery of Univerdidade Federal do Espírito Santo. The animals were placed in polypropylene cages with metal bars and wood shavings and they passed an acclimatization period of 7 days before the start of the experiments, with free access to standard commercial feed and water and they were kept under light/dark cycles of 12 h.

For the induction of solid tumors and evaluation of the antitumor activity of *C. jamacaru* extract, 200 µL of sarcoma 180 cells diluted in NaCl (0.9%) (5.10^5^ cells/mL) were injected, subcutaneously, in the dorsal region of the animals between the neck and the shoulder girdle. This procedure was conducted with 16 animals (*n* = 16). After the tumor induction protocol, the 16 animals were randomly separated into four treatment groups with four animals each (*n* = 4). A group of four animals that did not receive the sarcoma 180 cells (*n* = 4) was used as a healthy control.

All protocols involving animals were conducted according to the ethical principles of animal experimentation established by the Research Ethical Committee on Animal Use Univerdidade Federal do Espírito Santo (CEUA/UFES, certificate 89/2015).

#### 2.7.2. Selection of Doses and Treatment Groups

The treatment of the animals was started three days after tumor induction. The dosages chosen for the experiments were based on the LD_50_ of hydroalcoholic extract of *C. jamacaru* cladodes [22]. Three experimental groups of mice with sarcoma tumor received daily *C. jamacaru* extract i.p. at the dose of 5.0, 10.0 or 20.0 mg/kg b.w. (*C. jamacaru* treatment groups); a group of mice with sarcoma tumor received daily NaCl (0.9%) i.p (sarcoma group); and the group of healthy animals (without sarcoma tumor induction) received daily NaCl (0.9%) i.p (healthy group). The animals received the doses of *C. jamacaru* extract or NaCl (0.9%) for 20 consecutive days and 24 h after the last treatment the animals were euthanized by cervical dislocation.

#### 2.7.3. Tumor Inhibition

At the end of the experiment period, the tumors of the mice of each experimental group were removed. The tumors were weighed and the mean tumor weight of each experimental group was used to calculate the percentage of tumor inhibition by the formula:

$$(\%)\;\mathrm{Tumor}\;\mathrm{inhibition}=\frac{\left(\mathrm{Tumor}\;\mathrm{weight}\;\mathrm{in}\;A-\mathrm{Tumor}\;\mathrm{weight}\;\mathrm{in}\;B\right)}{\mathrm{Tumor}\;\mathrm{weight}\;\mathrm{in}\;A}\times 100$$

where “A” is the mean of the tumor weight of sarcoma group; “B” is the mean of the tumor weight of the *C. jamacaru* treatment groups.

#### 2.7.4. Macroscopic Analysis of Organs

Kidneys, liver, spleen and heart of animals from each experimental group were removed. The organs were evaluated for possible macroscopic abnormalities and subsequently weighed for comparison between the experimental groups.

#### 2.7.5. Micronucleus Test in Mice Peripheral Blood Cells

Three days after tumor induction, to assess the mutagenicity and cytotoxicity, peripheral blood was collected from mice prior to initiation of treatment (0 day of treatment) and at the end of treatment with NaCl (0.9%) or doses of *C. jamacaru* extract (20 days of treatment). The peripheral blood was obtained from the caudal artery of each animal of experimental groups using a sterile needle and the smears of whole blood were prepared on clean microscope slides, air dried. For each peripheral blood collection performed, two slides were prepared per animal, the cells were fixed in methanol (100%) and stained with Leishman twice in two different concentrations (100% for three minutes and 1 Leishman: 6 distilled water, for fifteen minutes) to differentiate immature polychromatic erythrocytes (PCE) and mature normochromatic erythrocytes (NCE), following the criteria described by Krishna and Hayashi [23]. The slides were analyzed under an optical microscope (Nikon Eclipse E200) with an increase of 1000 times. For the evaluation of mutagenicity, 2000 NCE were recorded per animal, 1000 NCE per slide, considering the micronucleated normochromatic erythrocytes (MNNCEs) [23]. The frequency of PCE in 2000 (PCE) per animal, 1000 NCE per slide, was used as a parameter of cytotoxicity [12].

### 2.8. Statistical Analysis

Data were evaluated a priori by normality test and the results were expressed as the mean ± standard deviation or median (Percentile 25—Percentile 75). *C. jamacaru* extract concentration required to reduce 50% of the DPPH, ABTS or Fe^2+^ chelating ions (EC_50_) and R^2^ were obtained by the linear curve, relating the antioxidant capacity of the extract and its concentrations. To evaluate the cytotoxicity of *C. jamacaru* extract *in vitro*, the cell viability of lymphocytes and sarcoma 180 were compared to the respective control cells by ANOVA *post hoc* Dunnett’s test (*p* < 0.05). The comparison between human lymphocytes and sarcoma 180 cells was performed by multiple *t* test (*p* < 0.05). For the evaluation of the anti-cytotoxicity of *C. jamacaru* extract *in vitro*, the lymphocytes cell viability was compared to the cisplatin treated cells by ANOVA *post hoc* Dunnett’s test (*p* < 0.05). To analyze the antitumor effect, mutagenicity, cytotoxicity and organs weight alterations on *in vivo* mice treatments, it was performed a comparison between the sarcoma group and experimental groups by Mann Whitney test (*p* < 0.05). Wilcoxon’s test (*p* < 0.05) was performed to evaluate mutagenicity and cytotoxicity *in vivo* prior to treatment initiation (0 day of treatment) and after treatment (20 days of treatment with NaCl (0.9%) or doses of *C. jamacaru* extract).

## 3. Results

### 3.1. Extract Yield and Phytochemistry

After 24 h in an oven dried at 50 °C were obtained 82.0 g of dried plant material. A total of 6.8 g of *C. jamacaru* hydroalcoholic crude extract was obtained, which corresponds to 8.29% of total extract yield. Preliminary phytochemistry of *C. jamacaru* extract showed positive reactions to coumarins, flavonoids and flavanol (cyanidin reaction) and TLC showed the presence of the alkaloid tyramine. The total flavonoid content was assayed by AlCl_3_ colorimetric method and determined that the crude extract of *C. jamacaru* cladodes contained 0.51 ± 0.14 µg/mL (rutin equivalent).

### 3.2. Evaluation of Antioxidant Activity

**Figure 1:**
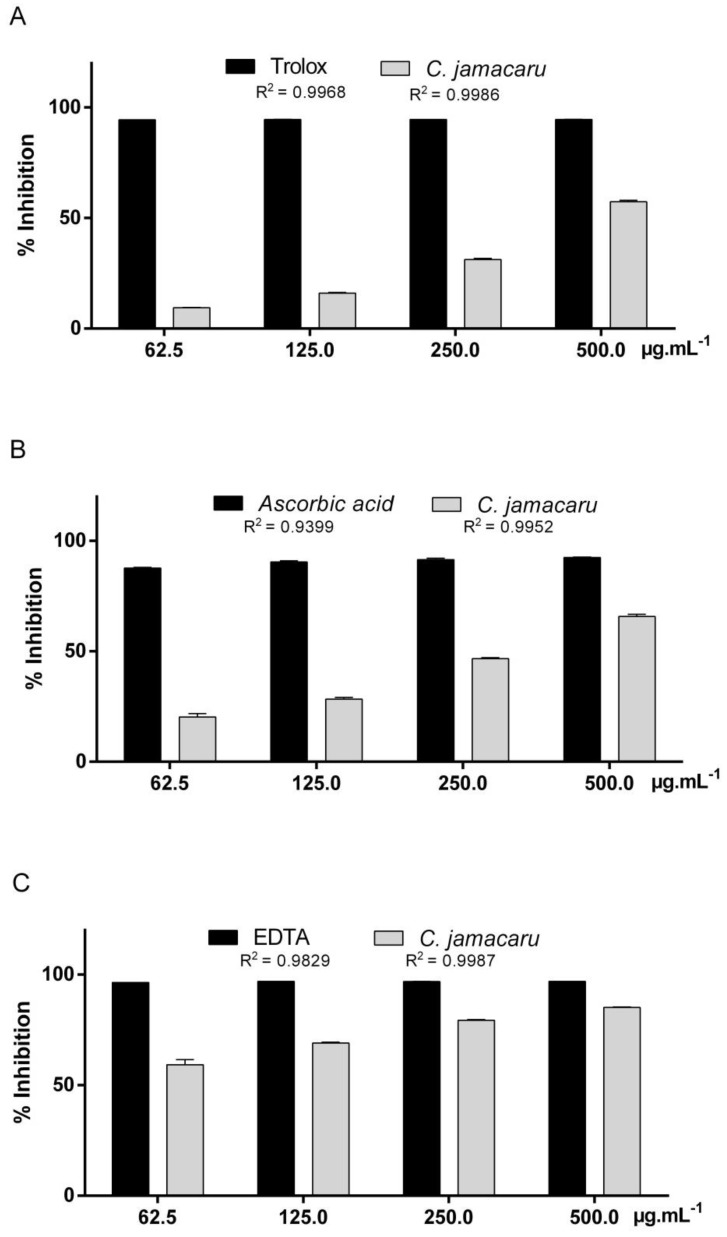
Antioxidant activity of *C. jamacaru* extract following DPPH, ABTS and Fe^2+^ chelation ions. (**A**) Antioxidant activity of *C. jamacaru* extract and of the Trolox standard shown by the percentage of inhibition of DPPH. (**B**) Antioxidant activity of *C. jamacaru* extract and of the ascorbic acid standard shown by the percentage of inhibition of ABTS. (**C**) Antioxidant activity of *C. jamacaru* extract and of the EDTA standard shown by the percentage in chelating activity on Fe^2+^ ions.

The results of the antioxidant activity of *C. jamacaru* extract are summarized in the Figure 1. In the DPPH assay, *C. Jamacaru* extract reached 9.43–57.36% of antioxidant activity and the standard trolox reached 94.42–94.60% (Figure 1A). Following the ABTS assay, the extract reached 20.25–65.76% of antioxidant activity and the standard ascorbic acid reached 87.66–92.86% (Figure 1B). In the chelating activity on Fe^2+^ ions, *C. jamacaru* extract reached 59.21–76.06% of antioxidant activity and the standard EDTA reached 96.47–96.92% (Figure 1C). The antioxidant activity of *C. jamacaru* extract expressed in half-maximal effective concentration (EC_50_) was: DPPH − EC_50_ = 427.74 ± 5.80 µg/mL, ABTS − EC_50_ = 270.57 ± 4.99 µg/mL, Fe^2+^ chelating ions − EC_50_ = 41.18 ± 7.59 µg/mL.

### 3.3. Cytotoxicity and Antiproliferative Activity In Vitro

In Table 1 the results of *C. jamacaru* extract cytotoxicity in human lymphocytes and sarcoma 180 cells are summarized.

*C. jamacaru* extract was able to reduce human lymphocyte cell viability after 24 h of exposure at 10.0 and 50.0 µg/mL and promote lymphocytes proliferation at 100.0 µg/mL. After 48 h of exposure, the lymphocytes cell viability at 10.0 µg/mL was reduced, at 50.0 µg/mL lymphocytes viability was statistically similar to the control and at 100.0 µg/mL *C. jamacaru* extract continued to promote lymphocytes proliferation. On the other hand, *C. jamacaru* extract reduced significantly the viability of sarcoma 180 cells after 24 h and after 48 h of exposure at all doses tested. When cytotoxicity of healthy and tumor cells was compared by multiple *t* test (*p* < 0.05), it was possible to infer that *C. jamacaru* extract was more cytotoxic to sarcoma 180 cells than to human lymphocytes at 24 h and 48 h.

### 3.4. Anti-Cytotoxic Activity In Vitro

Table 2 summarize the results of anti-cytotoxicity in the protocols of pre-treatment, simultaneous treatment and post-treatment with *C. jamacaru* extract. Following the pre-treatment protocol, compared to cisplatin-treated cells, it was observed that *C. jamacaru* extract increased cell viability of human lymphocytes at all tested doses. For the simultaneous treatment, it was observed statistical reduction of cisplatin induced-damage in cell viability at the treatment dose of 50.0 and 100.0 µg/mL. In the post-treatment protocol the treatment dose of 50.0 and 100.0 µg/mL reduced the damage induced for cisplatin. In all treatment protocol the dose of 100.0 µg/mL was the most effectively in cytotoxic damage inhibition.

### 3.5. Antitumor Activity In Vivo

Table 3 shows the results of antitumor activity induced by tested doses of *C. jamacaru* extract in mice with sarcoma. In comparison to sarcoma group, animals receiving *C. jamacaru* extract at the treatment dose of 20.0 mg/kg b.w. showed tumor reduction (86.07% of tumor inhibition). The treatment doses of 5.0 and 10.0 mg/kg b.w. were not able to induce a decrease in tumor weight.

### 3.6. Weight and Macroscopic Analysis of Organs

Macroscopic morphological abnormalities were not observed in the animal organs of the experimental groups. The weight of mice organs of each group is presented in Table 4. It was observed the reduction of the weight of the kidneys and heart of the animals treated with 5.0 and 10.0 mg/kg b.w. of *C. jamacaru* extract. The liver weight of the animals did not change and the spleen weight was increased in all animals with sarcoma compared to the healthy group of animals.

### 3.7. Mutagenicity and Cytotoxicity In Vivo

MNNCE and PCE frequency related to PCE in peripheral blood cells of rodents is summarized in Table 5. After tumor induction, at 0 day of treatment, MNNCE frequency before the start of treatments was increased in animals with sarcoma, indicating that the sarcoma tumor induction promotes mutagenic effects. However, at the end of treatment time (20 days of treatment), the frequency of MNNCE was not statistically different between the sarcoma group and the treatment groups, suggesting the absence of mutagenic damage. For the comparison over time (0 day vs. 20 days of treatment), no statistical difference was observed in the frequency of MNNCE.

Prior to initiation of treatment, at 0 day of treatment, the frequency of PCE per 1000 NCE was not significantly different between the experimental groups and the sarcoma group. After 20 days of treatment, in comparison to the group of healthy mice, there was no significant decrease in the frequency of PCE per 1000 NCE, suggesting the absence of cytotoxic damage. On the other hand, the frequency of PCE per 1000 NCE was increased in animals with sarcoma (sarcoma group) and treated with 5.0 mg/kg b.w. of *C. jamacaru* extract, suggesting alterations on mitotic cycle. Along treatment time, the comparison between 0 and 20 days of treatment showed a significant increase in PCE frequency in sarcoma group after 20 days of treatment (*p* = 0.0078). For the other treatment groups, no significant alterations were observed over time for the conditions tested.

## 4. Discussion

Biological active constituents present in natural products are not fully identified but due to their effectiveness, presumably minimal side effects and relatively low cost, their uses have increased [3]. A variety of bioactive compounds are found in natural products included in a healthy diet and they have been associated to the promotion of human health in the last decade [24]. In that way, plants of cactaceae family have been characterized as a feed source, as well as some studies evaluating cacti have demonstrated their uses as antioxidants and anticancer [25,26,27]. *Opuntia*, *Pereskia* and *Pilosocereus* are cacti genus that have been extensively studied and well documented for their antioxidant capacity and biological applications, however, there are few studies with *C. jamacaru*.

Phytochemicals are non-nutritive chemicals that occur naturally in plants and can be used in the plant defense mechanisms [28]. In the phytochemical analysis of *C. jamacaru* we detected coumarins, flavonoids (flavanol) and the alkaloid tyramine. Coumarin and flavonoids are phenolic compounds present in many plant foods and among polyphenolic substances, flavonoids are the class of compounds most commonly found in plants and can be classified into flavanols, flavanones, flavones, isoflavones, catechins, anthocyanins, proanthocyanidins and others [5]. On the other hand, alkaloids are nitrogen compounds found in medicinal plants that have been extensively studied and reported as antioxidants [29,30].

In our study, the total flavonoid content in hydroalcoholic extract of *C. jamacaru* cladodes was 0.51 µg/mL, similar to the observed in the study of De Sousa Araújo et al. [8], using methanolic extract of *C. jamacaru* cladodes (0.59 µg/mL). Both plants were collected in arid regions of Brazil and probably grown under similar light, water and nutrient availability. Figueroa-Cares et al. [31], in a study with cultivars, verified that flavonoid content may vary and suggests that the differences between flavonoid content in the cultivars can be related to the water scarcity and soil nutrients.

Alkaloids tyramine and N-methyltyramine are compounds produced by *C. jamacaru* and they are chemical markers of this specie [32]. Preliminary phytochemistry showed negative reaction to alkaloids by Dragendorff reagent and presented a positive reaction to the tyramine alkaloid by thin layer chromatography. Alkaloids are chemical compounds that have nitrogen atom in their structures. This group of chemicals is divided in true alkaloids, protoalkaloid, polyamine alkaloids, peptide and cyclopeptide alkaloids and pseudoalkaloids [33]. Dragendorff reagent has been used to detect tertiary and quaternary alkaloids [34,35,36]. Tyramine, as well as N-methylthyramine, are classified as primary alkaloids and were detected in our study by the specific protocol developed by Davet et al. [7]. The analysis suggests that the amount of tertiary and quaternary alkaloids in the extract of *C. jamacaru* was relatively low and not detected.

*C. jamacaru* extract showed antioxidant activity in all protocols tested (Figure 1). DPPH and ABTS assays are methods used to measure the ability of antioxidant to scavenging free radicals, which are the major factor in biological damages caused by oxidative stress and both radicals used in these assays are reduced to their stable or less reactive derivatives by the antioxidant compounds [37]. *C. jamacaru* hydroalcoholic extract was able to inhibit the activity of DPPH radicals (Figure 1A), EC_50_ = 427.74 µg/mL. The concentration of *C. jamacaru* extract required to achieve a 50% reduction in DPPH radicals was lower than the observed in MeOH and n-hexane fractions of cladodes extracts of the cactaceae *Opuntia monacantha*, EC_50_ = 833.0 and 469.0 µg/mL, respectively and larger than the observed in OM–EtOAc and OM–n-BuOH fractions of the same cactus extract, EC_50_ = 53.2 and 278.0 µg/mL, respectively [25]. For other cacti of the *Opuntia* genus, such as *O. dillenii*, the concentration required to reduce 50% of DPPH radicals was 48.0 µg/mL—EtOH 80% stem extract [38] and to the ethanolic extract of *O. ficus-indica* stem extract the EC_50_ was equivalent to 9.3 µg/mL [39].

ABTS assay showed better result than the observed in DPPH assay (Figure 1A,B). In a similar assay, *Pereskia bleo* aqueous extract showed a low antioxidant effect when compared to the ascorbic acid standard [40], different to that observed in our study. Added to this, the EC_50_ of *C. jamacaru* extract in ABTS test was 270.7 µg/mL, higher than the observed in *Pilosocereus gounellei* stem extract, EC_50_ = 62.4 µg/mL [9].

Ethanolic extracts of *Rumex vesicarius* leaves produced by three different extraction methods showed good antioxidant activity on chelating activity on Fe^2+^ ions assay, presenting EC_50_ between 157.4–185.3 µg/mL [41]. For the antioxidant assays performed in this study, the *C. jamacaru* extract presented better antioxidant activity in the chelating activity on Fe^2+^ ions assay, EC_50_ = 41.18 µg/mL. *C. jamacaru* extract presented in its composition alkaloids and phenolic compounds. In a study conducted by Klimaczewski et al. [30], the boldine alkaloid—commonly found in *Peumus boldus*—was able to induce good antioxidant activity in the DPPH assay but was not effective in promoting ion chelation in the Fe^2+^ ions. On the other hand, natural products rich in phenolics compounds have exhibited good ability to chelate ferrous ions, which has been related to the hydroxyl group on flavonoids [42]. Added to this, a study conducted with the alkaloid tyramine, compound detected in *C. jamacaru* extract, showed strong scavenging activity in DPPH assay and reducing power, reaching 86.34% of DPPH radical inhibition [43], which may have contributed to overall antioxidant activity.

The 3-[4-Dimethylthiazol-2-yl]-2,5-diphenyltetrazolium bromide (MTT) is a yellow tetrazolium salt, water soluble, that is converted to formazan, purple and insoluble in water. The conversion of MTT to formazan is mediated by the cleavage of the tetrazolium ring by succinate dehydrogenase within the mitochondria, as well as, it can be mediated by NADH or NADPH within the cells and out of mitochondria [44,45]. MTT assay is a cell viability assay used to determine cytotoxicity upon exposure to toxic substances and has been used to verify the viability of human lymphocytes [46,47] and sarcoma 180 cells exposed to natural products [48,49].

Neutral red, LDH leakage and the protein assays are also used to evaluate cell viability. However, neutral red and the MTT assay have been described as the most sensitive cytotoxicity assays and appear to be more sensitive in detecting early toxicity [44]. In this way, some compounds may inhibit mitochondrial respiration and induces active oxygen related cell death, generating reactive oxygen species within the mitochondria that promotes the damage mitochondrial components and therefore a cytotoxicity assay based on mitochondrial respiratory activity might be used to detect early toxicity following exposure to a mitochondrial toxicant [44].

We observed that *C. jamacaru* extract effectively reduced the viability of sarcoma cells 180 and that the extract did not induce significant reduction in the cellular viability of human lymphocytes, preventing and repairing cytotoxic damages (Table 1 and Table 2). These results suggest that *C. jamacaru* extract may promotes descytotoxic activity, acting directly on the cisplatin, inducing chemical or enzymatic inactivation of cytotoxic compounds (pre-treatment and simultaneous treatment protocols), as well as promoting bioanticytotoxic activity, inducing the repair process or acting on the processes that induce the cytotoxic damage (post-treatment protocol).

Cisplatin is a highly reactive molecule used for the treatment of cancers due to its ability to binds to RNA, DNA and proteins, forming different types of adducts and thus generate cytotoxic effects [50,51]. In particular, the adducts formed with nuclear DNA have been reported as key lesions that mediates the cytotoxic effect of cisplatin and the repair of these lesions may occur by intracellular DNA damage management pathways, such as nucleotide excision repair pathway, which plays a major role in removing cisplatin-nuclear DNA adducts [14,52].

Nuclear DNA damage is not sufficient to explain its use as effectiveness as an anticancer agent, since cisplatin toxicity does not depend only on the amount of drug accumulation in normal tissues, suggesting that nuclear DNA transcription blockage may not be the unique mechanism to determinate the toxic effect of cisplatin in non-replicating cells [14,53]. Cisplatin may accumulates into mitochondria, form adducts with mitochondrial DNA and proteins and increase intracellular ROS in normal cells [54,55,56,57,58].

The impairment of electron transport chain protein synthesis, as well as cisplatin-induced ROS generation, occur as consequence of its direct effect on mitochondrial DNA. Mitochondrial redox status, DNA integrity and bioenergetic functionality are also reported as key modulators of the cellular response to cisplatin-induced mitochondrial impairment and may be factors determining resistance to its cytotoxic effect [14]. Cisplatin exposure induces a mitochondria-dependent ROS response, which significantly contributes to cell death by enhancing the cytotoxic effect exerted through the formation of nuclear DNA damage [14]. Additionally, cisplatin promotes mitochondrial injury, energy imbalance and oxidative damage [14,59,60] and antioxidant compounds treatment may ameliorates the toxic effects promoted to cisplatin by increasing mitochondrial ROS scavenging.

Iron is related to tissue injury, ROS synthesis and reported as mediator of cisplatin tissue injury in cisplatin-induced nephrotoxicity [61,62]. This metal is reversibly oxidized and may generate powerful oxidant species, such as the hydroxyl radical (Haber-Weiss reaction), or generates highly reactive iron-oxygen complexes such as ferryl or perferryl ions [61], increasing the damage induced by cisplatin. On the other hand, the treatment with both iron chelators and hydroxyl radical scavengers may prevent cisplatin cytotoxicity and renal failure [62], which suggests that compounds capable of chelating iron ions may reduce cisplatin-induced damage.

*In vivo* anticancer activity of *C. jamacaru* extract has already been reported in the literature. In the study conducted by Souza et al. [22], after the induction of sarcoma, male mice received daily doses of hydroethanolic extract of *C. jamacaru* cladodes at dose of 40 mg/kg b.w. and exhibited 65.61% tumor inhibition. Similar to the study of Souza et al., we demonstrated in our study the ability of the *C. jamacaru* hydroalcoholic extract to promote antitumor activity against sarcoma *in vivo* (86.07% tumor inhibition).

In our study, no macroscopic abnormalities were observed, kidney and heart weight decreased in animals receiving *C. jamacaru* extract doses of 5.0 and 10.0 mg/kg b.w. and, compared with the healthy group of animals, the weight of the spleen was increased in all animals with sarcoma. No macroscopic or histopathological abnormalities have been described in healthy rats receiving doses of ethanolic extract of cladodes of *C. jamacaru* for 30 days, as well as alteration in kidney, liver, spleen and heart weight in relation to body [63].

Micronucleus test in peripheral blood can be performed in different time points along treatment time and allows to verify, through the differentiation between polychromatic and normochromatic erythrocytes, both mutagenicity and cytotoxicity [12]. In an experiment conducted with pregnant rats treated with of methanolic extract of *C. jamacaru*, Messias et al. [64] report that the extract of this cactus did not induce cytotoxic and mutagenic damages and was able to promote mild antimutagenic effect. Our findings suggest that sarcoma induction promotes mutagenic damage, detected three days after the induction of solid tumors (0 day of treatment). However, at the end of the treatment time (20 days of treatment) this effect is not observed, suggesting the absence of mutagenic damage over time. In addition, at the end of the treatment time, it was observed that the presence of solid tumors of sarcoma led to the increase of PCE frequency, indicating changes in the mitotic cycle that may lead to increase of PCE proliferation.

There are several mechanisms involved in the evolution of a normal cell into a potentially malignant cell and most of them interfere in cell division [65]. In this way, knowledge of the cell cycle and its mechanisms are important tools for understanding the etiology of cancer [66]. The drugs used in cancer treatment may have action on tumor cells that are in the cell cycle (specific cell cycle drugs) or act on tumor cells regardless of whether they are traversing the cycle or being resting in the G_0_ compartment (non-specific cell cycle drugs) [67,68,69].

Natural products with specific cell cycle action are used as antineoplastic agents. Among the natural cytotoxic products used clinically in the treatment of neoplasias are plant alkaloids, nitrogenous compounds biosynthesized from amino acids, such as vinblastine and vincristine (promote inhibition of mitotic spindle, binding to the microtubular proteins and interrupting cell division in metaphase) [70]; taxol (inhibits mitotic spindle, induces tubulin dimerization and stabilization of tubules, protecting them from depolymerization, leading to blocking of multiplication and loss of cell viability) [70]; and podophyllotoxins or epipodophyllotoxins (block the cells in the S and G_2_ phases and inhibit the action of the enzyme topoisomerase II, leading to DNA damage) [67,69,70].

## 5. Conclusions

*C. jamacaru* extract presented in its chemical composition phenolic and nitrogen compounds and good antioxidant ability to chelate metallic ions. We suggest that the increased ability of *C. jamacaru* extract in chelating metals may be related to anti-cytotoxic activity against cisplatin *in vitro*. In addition, we suggest that *C. jamacaru* extract selectively acts on tumor cells, probably blocking metabolic processes to the survival of the cells, acting on cell cycle of the tumor cells *in vitro* and *in vivo*, leading to anticancer effects and tumor reduction. However, additional studies are necessary to determine the mechanisms involved in the anti-cytotoxic and anticancer effects. These findings reinforce that this cactus used in human food also presents great therapeutic potential and can be used for drug discovery.

## Figures and Tables

**Table 1:** *In vitro* cytotoxicity of *C. jamacaru* extract (10.0, 50.0 or 100.0 µg/mL) in human lymphocytes and sarcoma180 cells by MTT assay.

Treatment	Cell Viability (%) ± SD							
	24 h of Treatment				48 h of Treatment			
	Lymphocyte	*p*	Sarcoma-180	*p*	Lymphocyte	*p*	Sarcoma-180	*p*
Control	100.00 ± 2.41	–	100.00 ± 0.99	–	100.00 ± 4.83	–	100.00 ± 3.48	–
*C. jamacaru* 10.0 µg/mL	81.08 ± 1.16 ****^†^	<0.0001	30.53 ± 3.51 ^####†^	<0.0001	84.40 ± 1.58 ***^†^	0.0003	16.09 ± 0.17 ^####†^	<0.0001
*C. jamacaru* 50.0 µg/mL	88.80 ± 1.34 **^†^	0.0043	29.84 ± 1.18 ^####†^	<0.0001	95.41 ± 0.80 ^†^	0.4243	17.54 ± 0.44 ^####†^	<0.0001
*C. jamacaru* 100.0 µg/mL	122.39 ± 5.47 ****^†^	<0.0001	19.82 ± 1.60 ^####†^	<0.0001	133.95 ± 7.07 ****^†^	<0.0001	22.29 ± 1.10 ^####†^	<0.0001

The values are the means  ±  SD. Cell viability was compared to its respective control cells by ANOVA *post hoc* Dunnett’s test—** *p* < 0.01, *** *p* < 0.001 or **** *p* < 0.0001 vs. human lymphocytes control; ^####^ *p* < 0.0001 vs. sarcoma 180 control. The comparison between the human lymphocytes and sarcoma 180 cells was performed by multiple *t* test—^†^ *p*<0.05.

**Table 2:** *In vitro* anti-cytotoxicity of *C. jamacaru* extract (10.0, 50.0 or 100.0 µg/mL) in human lymphocytes cells by MTT assay.

Treatment	Cell Viability (%) ± SD								
	Pre-Treatment	*p*	% Reduction	Simultaneous Treatment	*p*	% Reduction	Post-Treatment	*p*	% Reduction
Control	100.00 ± 4.83 *	0.0182	–	100.00 ± 2.4 ***	0.0006	–	100.00 ± 4.83 ***	0.0008	–
Cisplatin	73.85 ± 6.50	–	–	74.52 ± 2.68	–	–	73.85 ± 6.50	–	–
*C. jamacaru* 10.0 µg/mL + Cisplatin	89.44 ± 5.84 *	0.0201	59.62	79.15 ± 2.41	0.6822	20.27	76.15 ± 4.42	0.9635	8.80
*C. jamacaru* 50.0 µg/mL + Cisplatin	96.79 ± 2.11 *	0.0367	87.72	87.65 ± 3.54 *	0.0421	52.77	90.82 ± 2.75 *	0.0147	64.89
*C. jamacaru* 100.0 µg/mL + Cisplatin	127.06 ± 4.83 ***	0.0001	>100.00	106.57 ± 10.62 ***	0.0001	>100.00	115.60 ± 8.38 ****	<0.0001	>100.00

The values are the means  ±  SD. Cell viability was compared to the cisplatin treated cells in each treatment protocol by ANOVA *post hoc* Dunnett’s test—* *p* < 0.05, ** *p* < 0.01, *** *p* < 0.001 or **** *p* < 0.0001 vs. human lymphocytes control.

**Table 3:** Tumor weight of *Swiss* albino mice sarcoma induced treated with *C. jamacaru* extract (5.0, 10.0 or 20.0 mg/kg b.w.).

Treatment	Tumor Weight (P_25_–P_75_)	*p*	% Tumor Inhibition
Sarcoma + NaCl (0.9%)	0.070 (0.038–0.210)	–	–
Sarcoma + *C. jamacaru* 5.0 mg/kg b.w.	0.130 (0.010–0.610)	0.8571	–
Sarcoma + *C. jamacaru* 10.0 mg/kg b.w.	0.130 (0.048–0.430)	0.5606	–
Sarcoma + *C. jamacaru* 20.0 mg/kg b.w.	0.015 (0.012–0.023) *	0.0238	86.07

The values are median (Percentile 25—Percentile 75). P_25_ = Percentile 25; P_75_ = Percentile 75. Tumor weight of *C. jamacaru* treatment groups were compared to the sarcoma group by Mann Whitney test (*p* < 0.05)—* *p* < 0.05 vs. tumor weight of sarcoma group.

**Table 4:** Organs weight of *Swiss* albino mice sarcoma induced treated with *C. jamacaru* extract (5.0, 10.0 or 20.0 mg/kg b.w.).

Treatment	Weight (g) (P_25_–P_75_)							
	Kidney	*p*	Liver	*p*	Spleen	*p*	Heart	*p*
Sarcoma + NaCl (0.9%)	0.690 (0.628–0.713)	–	2.510 (2.460–3.043)	–	0.270 (0.233–0.415)	–	0.245 (0.228–0.300)	–
Sarcoma + *C. jamacaru* 5.0 mg/kg b.w.	0.575 (0.443–0.655) *	0.0429	2.495 (1.978–2.653)	0.5619	0.260 (0.228–0.353)	0.8048	0.205 (0.140–0.225) *	0.0286
Sarcoma + *C. jamacaru* 10.0 mg/kg b.w.	0.530 (0.480–0.570) *	0.0047	2.400 (1.910–2.580)	0.3625	0.250 (0.200–0.310)	0.4219	0.190 (0.150–0.220) **	0.0047
Sarcoma + *C. jamacaru* 20.0 mg/kg b.w.	0.630 (0.535–0.688)	0.1810	2.715 (2.405–2.905)	>0.9999	0.285 (0.240–0.335)	0.8048	0.205 (0.190–0.235)	0.0524
Health + NaCl (0.9%)	0.560 (0.505–0.668)	0.0762	2.290 (1.895–2.663)	0.1238	0.170 (0.108–0.225) *	0.0381	0.170 (0.145–0.240)	0.1095

The values are median (Percentile 25—Percentile 75). P_25_ = Percentile 25; P_75_ = Percentile 75. Organs weight of experimental groups were compared to the sarcoma group by Mann Whitney test (*p* < 0.05)—* *p* < 0.05 or ** *p* < 0.01 vs. organ weight of sarcoma group.

**Table 5:** Frequency of micronucleated normochromatic erythrocytes (MNNCE) and polychromatic erythrocytes (PCE) in 1000 NCE in whole peripheral blood of *Swiss* albino mice sarcoma induced treated with *C. jamacaru* extract (5.0, 10.0 or 20.0 mg/kg b.w.).

Treatment	MNNCE/1000 NCE (P_25_–P_75_)				PCE/1000 NCE (P_25_–P_75_)			
	0 Day of Treatment	*p*	20 Days of Treatment	*p*	0 Day of Treatment	*p*	20 Days of Treatment	*p*
Sarcoma + NaCl (0.9%)	5.50 (3.25–6.75)	–	3.00 (0.25–4.75)	–	7.50 (4.00–21.75) ^†^	–	23.50 (16.00–38.00) ^†^	–
Sarcoma + *C. jamacaru* 5.0 mg/kg b.w.	5.50 (3.25–6.75)	>0.9999	1.50 (0.25–3.50)	0.6246	7.50 (4.00–21.75)	>0.9999	16.50 (4.75–21.75)	0.0870
Sarcoma + *C. jamacaru* 10.0 mg/kg b.w.	5.50 (3.25–6.75)	>0.9999	4.50 (2.25–5.25)	0.4272	7.50 (4.00–21.75)	>0.9999	8.50 (6.25–17.00) ^#^	0.0263
Sarcoma + *C. jamacaru* 20.0 mg/kg b.w.	5.50 (3.25–6.75)	>0.9999	2.50 (2.00–3.00)	0.9459	7.50 (4.00–21.75)	>0.9999	10.00 (4.50–14.25) ^##^	0.0093
Health + NaCl (0.9%)	0.00 (0.00–1.75) **	0.0031	2.50 (1.25–3.75)	0.7120	12.00 (7.75–27.75)	0.2890	9.50 (5.00–13.50) ^##^	0.0093

The values are median (Percentile 25—Percentile 75). P_25_ = Percentile 25; P_75_ = Percentile 75. MNNCE or PCE frequency in 1000 NCE were compared to the sarcoma group by Mann Whitney test—** *p* < 0.01 vs. MNNCE frequency per 1000 NCE of sarcoma group; ^#^ *p* < 0.05 or ^##^ *p* < 0.01 vs. PCE frequency per 1000 NCE of sarcoma group. The comparison of MNNCE or PCE frequency per 1000 NCE at 0 and 20 days of treatment was performed by Wilcoxon test—^†^ *p* < 0.01.
